# Comparative Cytological and Transcriptome Analysis Revealed the Normal Pollen Development Process and Up-Regulation of Fertility-Related Genes in Newly Developed Tetraploid Rice

**DOI:** 10.3390/ijms21197046

**Published:** 2020-09-24

**Authors:** Jinwen Wu, Yuanmou Chen, Hong Lin, Yang Chen, Hang Yu, Zijun Lu, Xiang Li, Hai Zhou, Zhixiong Chen, Xiangdong Liu

**Affiliations:** 1 State Key Laboratory for Conservation and Utilization of Subtropical Agro-Bioresources, South China Agricultural University, Guangzhou 510642, China; jwwu@scau.edu.cn (J.W.); chymou@stu.scau.edu.cn (Y.C.); nxylh@stu.scau.edu.cn (H.L.); cy530871943@stu.scau.edu.cn (Y.C.); hyu@stu.scau.edu.cn (H.Y.); zjlu@stu.scau.edu.cn (Z.L.); xiangli@sgu.edu.cn (X.L.); haizhou@scau.edu.cn (H.Z.); chenzx@scau.edu.cn (Z.C.); 2 Guangdong Provincial Key Laboratory of Plant Molecular Breeding, South China Agricultural University, Guangzhou 510642, China; 3 College of Agriculture, South China Agricultural University, Guangzhou 510642, China; 4 Guangdong Laboratory for Lingnan Modern Agriculture, South China Agricultural University, Guangzhou 510642, China

**Keywords:** new tetraploid rice, pollen fertility, polyploidy, transcriptome analysis

## Abstract

Autotetraploid rice is a useful germplasm for polyploid rice breeding; however, low seed setting is a major hindrance for its utilization. Here, we reported the development of a new tetraploid rice, Huoduo1 (H1), which has the characteristic of high fertility, from crossing generations of autotetraploid rice. Cytological observations displayed the high fertility of the pollen (95.62%) in H1, a lower percentage of pollen mother cell (PMC) abnormalities, and stable chromosome configurations during the pollen development process compared with its parents. Using RNA-seq analysis, we detected 440 differentially expressed genes (DEGs) in H1 compared with its parents. Of these DEGs, 193 were annotated as pollen fertility-related genes, and 129 (~66.8%) exhibited significant up-regulation in H1 compared with the parents, including three environmentally sensitive genic male sterility genes (*TMS9-1*, *TMS5*, and *CSA*), one meiosis gene (*RAD51D*), and three tapetal-related genes (*MIL2*, *OsAP25*, and *OsAP37)*, which were validated by qRT-PCR in this study. Two genes, *TMS9-1* and *TMS5*, were knocked out using CRISPR/Cas9 technology, and their mutants displayed low fertility and the abnormal development of pollen. Our findings provide evidence for the regulatory mechanisms of fertility in tetraploid rice and indicated that the up-regulation of pollen fertility-related genes may contribute to the high fertility in new tetraploid rice.

## 1. Introduction

Polyploidy is one of the motivations in biological evolution, and it prevalently occurs in the plant evolution process [1]. Approximately 70% of plants have experienced at least one polyploidy event during their evolutionary history [2]. *S*everal advantages, including greater variation, high biomass yield, and resistance to insect pests and diseases, are found in polyploidy species compared with their original species [3,4]. Two categories of polyploidy plants, autopolyploidy and allopolyploidy species, generally exist in nature [5]. In contrast to the higher attraction of allopolyploidy plants, little is known regarding the real appearance of autotetraploid plants in nature despite potential weaknesses, such as meiotic instability and reduced fertility. Increasing evidence indicates that the real appearance of autotetraploid plants in nature might be significantly underestimated [6].

Autotetraploid rice is a useful germplasm derived from diploid rice by chromosome doubling. In comparison with corresponding rice, stronger biological vigor and heterosis were found in autotetraploid rice [7,8,9]; however, low pollen fertility is a major hindrance for its utilization [10,11]. Pollen abnormalities appear to be the major obstacles for a normal seed set [8,10]. Several previous studies have focused on the causes of low pollen fertility in autotetraploid rice, and the results were mainly focused on the abnormal pollen development process [10,12,13,14,15,16,17]. Abnormal chromosome behavior during meiosis and the down-regulation of pollen fertility genes were the primary causes of pollen sterility in autotetraploid rice. Therefore, it is critical to reveal the reasons for low pollen fertility and to acquire high-pollen-fertility tetraploid materials.

After years of efforts, we successfully developed new “autotetraploid rice lines” by selective breeding and crossing of successive generations [18,19,20]. The new “autotetraploid rice” displayed high fertility (>80%) and high heterosis when crossed with other autotetraploid rice lines with low fertility [11,18,19]. The F_2_ and F_3_ populations also displayed high fertility and stable morphological traits, for instance, neo-*Arabidopsis* [18,19,21]. Notably, the new “autotetraploid rice” is not an allotetraploid rice; however, its chromosome behavior is nearly normal, which contributed to high fertility and specific DNA mutations that were different from those of autotetraploid rice [22]. Neo- tetraploid rice is a tetraploid rice line with normal fertility and is a key step to overcome the sterility of F_1_ hybrids in tetraploid rice. Our group has reported three neo-tetraploid rice materials that could overcome the sterility of autotetraploid rice and produce high heterosis [18,19]. However, little is known regarding the complex regulatory mechanisms of heterosis and fertility in neo-tetraploid rice.

High-throughput technologies, such as transcriptome analysis, can provide useful insights for detecting genetic regulation in rice pollen development. A large number of RNA sequencing datasets are publicly available on TIGR (The Institute for Genomic Research Rice Genome Annotation) and GEO (Gene Expression Omnibus) [23,24]. Reliable pollen development networks and novel pollen development-related genes could be used to analyze the characteristics of genetic regulation in the pollen development process. There are several studies that focused on the pollen development process in autotetraploid rice [10,11,15]. In the autotetraploid rice Taichung65-4x, 124 and 6 genes were detected as up- and down-regulated, respectively, in Taichung65-4x compared with its corresponding rice during the pollen development stage. In the autotetraploid rice T449-4x, a total of 75 meiosis-related genes displayed differential expressions compared with diploid rice during the meiosis stage [11]. In the autotetraploid rice 02428-4x, 122 genes were identified that might be associated with low pollen fertility during pollen development [15]. Guo et al. (2017) revealed significant variations in new tetraploid rice and its two parents, and 42 meiosis stage-specific genes or meiosis-related genes were detected by transcriptome analysis [18]. All of these transcriptome data provide the possibility to reveal the genetic regulation of high pollen fertility in new tetraploid rice.

New tetraploid rice is thought to be an effective way to overcome the low fertility of autotetraploid rice; therefore, understanding the mechanism of fertility regulation in new tetraploid rice is important. In this study, we developed a new tetraploid rice, named Huaduo1 (H1), which was registered (Protection for New Varieties of Plants in China in 2016). We used cytological analysis and RNA-sequencing analysis to analyze the mechanism of high pollen fertility in new tetraploid rice with respect to that of its two parents. Cytological analysis was used to compare the phenotypic differences between the H1 and its parents. Transcriptional analysis was used to discover the large number of pollen fertility-related differentially expressed genes (DEGs) resulting in the high fertility in H1. We selected the representative genes to verify the relationship between the up-regulated pollen fertility genes and the high fertility in H1 and its parents. The results of this study may help us to understand the molecular mechanism of high fertility in new tetraploid rice.

## 2. Results

### 2.1. Breeding Procedure of New Tetraploid Rice Huaduo1

**Figure 1:**
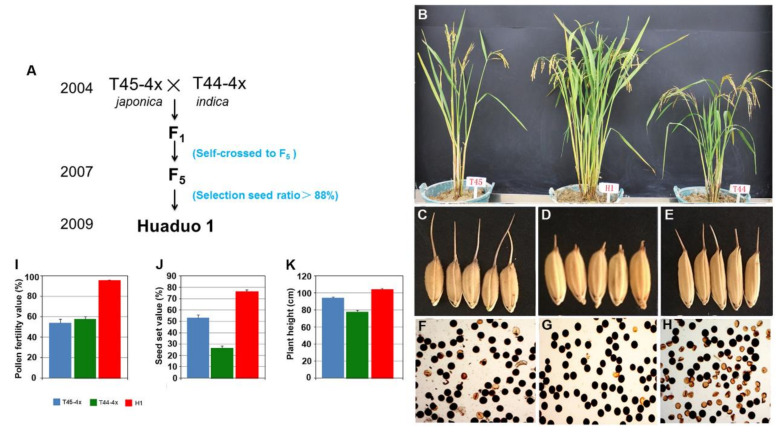
Breeding procedure and phenotype of Huaduo1 (H1) and its parents. (**A**) Breeding procedure of H1. (**B**) Morphologies of the whole plant of H1 and its two parents. (**C**–**E**) Grain size of H1 and its two parents. (**F**–**H**) Pollen grains stained with I_2_-KI in H1 and its two parents. (**I**–**K**) Comparison of the pollen fertility (**I**), seed set ratio (**J**), and plant height (**K**) between H1 and its two parents.

A hybrid of two autotetraploid rice plants, Jackson-4x (T45-4x) and 96025-4x (T44-4x), was generated in 2004, and the F_1_ hybrid plants were harvested and continuously self-crossed until F_5_ in 2007 (Figure 1). One line with more than 80% seed setting was found in that year, and a new tetraploid rice line, Huaduo1 (H1), was developed in 2009 and registered for PVP (Protection for New Varieties of Plants) in China in 2016 (Figure 1). Huoduo1 displayed significant differences in agronomic traits compared with its parent, which included high pollen fertility (95.62%) and seed setting (80%) (Figure 1; Table 1 and Table 2). H1 also showed significant heterosis in yield-related traits, including the number of filled seeds, 1000-seed weight, and seed setting when it was crossed with autotetraploid rice (Table S1).

To evaluate the ability to overcome the sterility of hybrids in H1, we developed hybrids with gene interactions in the pollen sterility loci, *Sa*, *Sb*, and *Sc,* using H1 crossed with Taichung 65-4x and its pollen sterility isogenic lines (Table S2). All of the hybrids had high seed setting (>80%) with gene interactions in the pollen sterility loci, *Sa*, *Sb*, and *Sc* (Table S2), suggesting that H1 may have neutral pollen fertility genes that could overcome the sterility of hybrids. All of these results indicate that H1 exhibited significant phenotypic variation compared with its two parents and has the potential to overcome the sterility of autotetraploid rice hybrids.

### 2.2. Comparison of Anther Development between Huaduo1 and Its Parents

**Figure 2:**
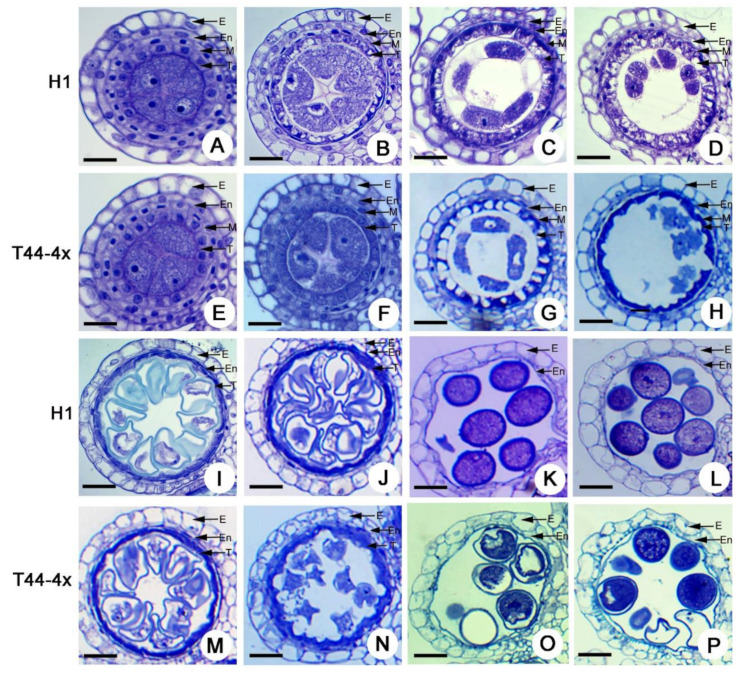
Semi-thin sections of H1 and its low-pollen-fertility parent T44-4x anthers. A to D, I to L Semi-thin sections of H1 anthers. (**A**) pre-meiotic interphase; (**B**) meiosis stage I; (**C**) meiosis stage I; (**D**) meiosis stage II; (**I**) single microspore stage; (**J**) late bicellular stage; (**K**) mature pollen stage; (**L**) mature pollen stage. E to H, M to P Semi-thin sections of low-pollen-fertility parent T44-4x anthers. (**E**) pre-meiotic interphase; (**F**) meiosis stage I; (**G**) meiosis stage I; (**H**) meiosis stage II; (**M**) single microspore stage; (**N**) late bicellular stage; (**O**) mature pollen stage; (**P**) mature pollen stage. E, En, M, and T indicate the epidermis, endothecium, middle layer, and tapetum, respectively. Bars = 50 μm.

To evaluate the variation of pollen fertility between H1 and its parents, we observed the pollen development of H1 and its low fertility parent T44-4x. The results indicated that the anther development process of H1 was similar to that of its low fertility parent T44-4x (Figure 2).

Anther development in H1 was primarily divided into eight differential stages: pollen mother cell formation (Figure 2A), meiosis (Figure 2B–D), early microspore, middle microspore (Figure 2I), late microspore, early bicellular, late bicellular (Figure 2J), and mature pollen (Figure 2K,L).

In H1, a four-layer anther wall (from the outside to the inside: epidermis, endothecium layer, middle layer, and tapetum) was generated at the pollen mother cell formation stage (Figure 2A). No obvious defects were found between the H1 and T44-4x anthers in the formation of PMCs (Figure 2B,F). During pollen mother cell (PMC) meiosis, the PMCs underwent meiosis, and normally formed dyads with cell plates in H1 and T44-4x were clearly observed (Figure 2C,G). Subsequently, dyads and tetrads also formed in H1 and T44-4x, and no obvious abnormalities were found in this stage (Figure 2D,H). Thereafter, the microspores of H1 underwent vacuolation and mitosis to form mature pollen with spherical or elliptical shapes (Figure 2I–L). In contrast, the microspores of T44-4x degraded further after the late microspore stage (Figure 2M,N), with typical abortion at the mature pollen stage, which resulted in abortive pollens (Figure 2O,P). All these results suggest that H1 caused slight defects in the microspores compared with T44-4x during the pollen development process.

### 2.3. Comparison of Chromosomal Behavior between Huaduo1 and Its Parents

**Figure 3:**
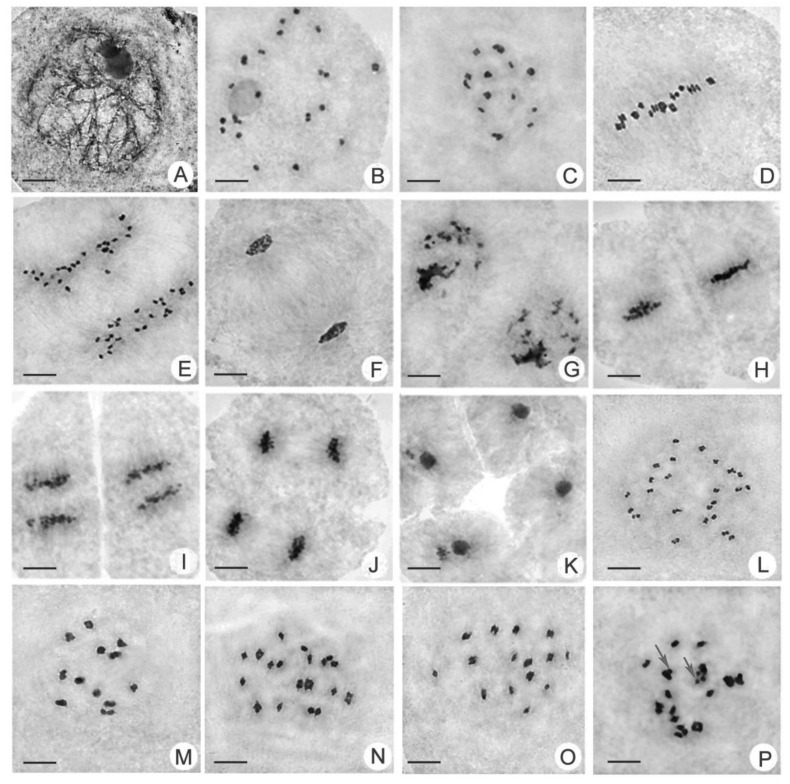
Chromosome behaviors and chromosome configurations in H1 and its parents during PMC meiosis (×3000). (**A**) Zygotene. (**B**) Diakinesis. (**C**) Diakinesis. (**D**) Metaphase I. (**E**) Anaphase I. (**F**) Telophase I. (**G**) Prophase II. (**H**) Metaphase II. (**I**) Anaphase II. (**J**) Telophase II. (**K**) Tetrad stage. (**L**) Diakinesis, 24 II. (**M**) Diakinesis, 12 IV. (**N**) Diakinesis, 7 IV+ 10II. (**O**) Diakinesis, 9 IV+ 6II. (**P**) Diakinesis, univalent (arrow). Bars = 10 μm.

**Figure 4:**
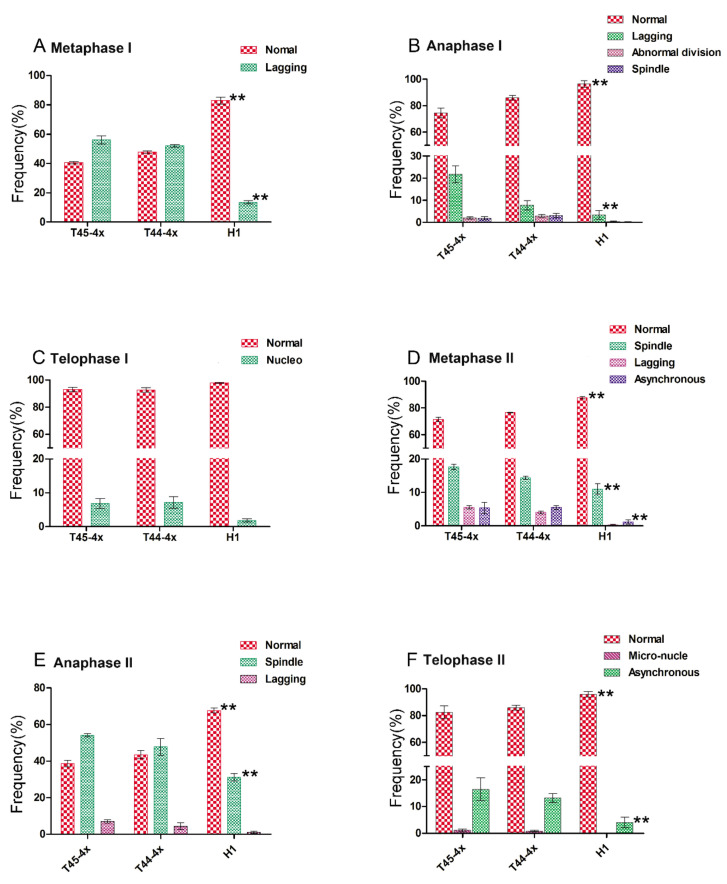
Frequency of PMCs in H1 compared with its parents at the meiosis stage. (**A**) Frequency of normal cells and main type of abnormal cells at Metaphase I. (**B**) Frequency of normal cells and main type of abnormal cells at Anaphase I. (**C**) Frequency of normal cells and main type of abnormal cells at Telophase I. (**D**) Frequency of normal cells and main type of abnormal cells at Metaphase II. (**E**) Frequency of normal cells and main type of abnormal cells at Anaphase II. (**F**) Frequency of normal cells and main type of abnormal cells at Telophase II. **, indicates a significant difference between H1 and its two parents.

For the important role of meiosis in autotetraploid rice during the pollen development process, we also observed the chromosomal behavior of PMCs in H1 and its parents. Similar meiotic processes and stage divisions were found in H1 and its parents, which is consistent with our previous study (Figure 3). A total of six key meiosis stages—Metaphase I, Anaphase I, Telophase I, Metaphase II, Anaphase II, and Telophase II—were observed, and the percentage of abnormalities is summarized (Figure 4; Tables S3 and S4).

H1 showed a lower percentage of abnormal PMCs compared with its two parents (Figure 4). In this study, the percentages of abnormal cells in H1 were 19.48%, 1.61%, 1.80%, 13.64%, 34.29%, and 1.94% in Metaphase I, Anaphase I, Telophase I, Metaphase II, Anaphase II, and Telophase II, respectively (Tables S3 and S4). In contrast, the autotetraploid rice parents of T45-4x and T44-4x showed many more abnormalities than H1 (Figure 4). For example, the percentages of abnormal cells in T44-4x were 52.14, 15.05, 3.96, 23.10, 52.14, and 12.12% in Metaphase I, Anaphase I, Telophase I, Metaphase II, Anaphase II, and Telophase II, respectively (Tables S3 and S4).

Additionally, chromosome configurations between H1 and its two parents were significantly different (Figure 3). Higher percentages of quadrivalent and bivalent configurations were frequently observed in H1 relative to those in its two parents (Table S5). However, chromosome configurations in T45-4x and T44-4x exhibited a much more complicated pairing style, such as univalent, trivalent, and other types of multivalent styles (Table S5). All of these results indicate that H1 had a higher percentage of normal cells and more stable chromosome configurations than those of the parents during meiosis.

### 2.4. Comparative Transcriptome Analysis of Huaduo1 and Its Parents in Meiosis

Transcriptome analysis was further conducted to explore the possible variations of the gene expression level associated with high pollen fertility seen in H1. We collected the meiotic anthers of H1 and its parents and detected their gene expression to identify the possible DEGs of high pollen fertility. From this study, we also divided the DEGs into three groups based on their origin derived from the two parents or non-parental variations. Group I was termed for the DEGs between H1 and its parent T44-4x (Table S6a). Group II was termed for the DEGs between H1 and its parent T45-4x (Table S6b). Group III was termed for the common DEGs that were differentially expressed in both H1 and its two parents, T44-4x and T45-4x (Table S6c).

**Figure 5:**
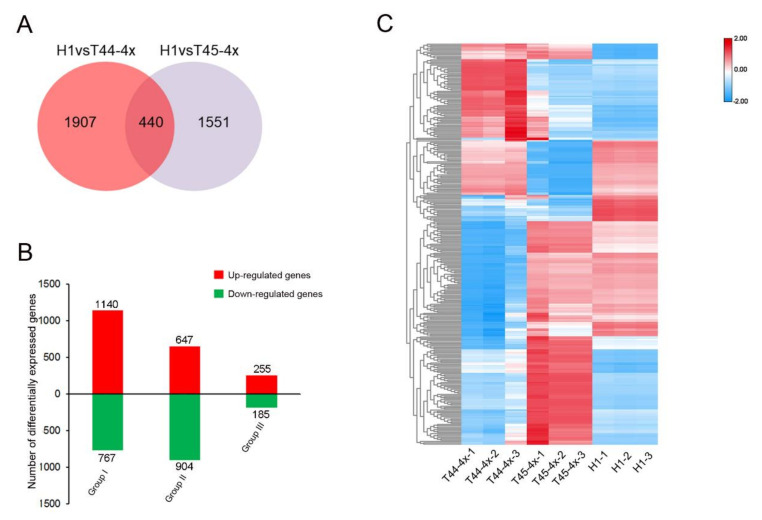
Differentially expressed genes in meiotic anthers of H1 detected by RNA-sequencing analysis compared to its two parents. (**A**) Venn diagram of differentially expressed genes in H1 detected by RNA-sequencing analysis. (**B**) Number of differentially expressed genes in H1 compared to its parents. Group I refers to the DEGs of H1 derived from T44-4x; Group II refers to the DEGs of H1 derived from the T45-4x; Group III refers to differentially expressed genes in H1 and its two parents. (**C**) Expression patterns of common DEGs in H1 and its two parents. Red and blue colors indicate differentially expressed genes that were up- and down-regulated, respectively.

Using these comparison groups, we identified a total of 3896 genes that were differentially expressed (twofold at a *p* value of <0.05) between H1 and its parents in meiosis (Figure 5A). Among these DEGs, 1140 and 767 genes were up- and downregulated, respectively, in Group I, (Figure 5B; Table S6a). We found 647 and 907 genes that were up- and down-regulated, respectively, in Group II (Figure 5B; Table S6b). Additionally, a total of 440 genes were detected and found to be differentially expressed in H1 compared with its two parents. Indeed, 255 and 185 genes were found to be up- and down-regulated, respectively (Figure 5B; Table S6c). We then focused on these common DEGs which were both differentially expressed in H1 and its two parents. We categorized both the up- and downregulated genes using Cluster 3.0 software and obtained an overview of the transcriptome relationships (Figure 5C).

Gene ontology (GO) analysis was conducted to annotate the common up- and down- regulated DEGs between H1 and its parents (Figure S1). The GO enrichment classification suggested that the genes from the biological process, cellular component, and molecular function categories showed significant variation. In the biological process category, six prominent functional gene classes—cellular process, transport, transcription, reproductive process, reproduction and pollination—were over-represented in the up-regulated gene classes. Additionally, response to stimulus, response to stress, and response to endogenous stimulus were over-represented in the down-regulated gene classes (Figure S1A). In the cellular component category, three prominent functional gene classes, nucleus, integral to membrane, and intrinsic to membrane, were over-represented in the upregulated gene classes, and four prominent functional gene classes, extracellular region, cell wall, external encapsulating structure, and thylakoid, were over-represented in the downregulated gene classes (Figure S1B). In the molecular function category, four prominent functional gene classes—transcription regulator activity, DNA binding, transporter activity, and cation binding—were over-represented in the up-regulated gene classes, and four prominent functional gene classes—catalytic activity, ion binding, oxidoreductase activity, and nucleotide binding—were over-represented in the down-regulated genes (Figure S1C). All of these results indicate that the upregulated genes were mainly involved in transport, transcription regulator activity, and reproduction. Alternatively, the down-regulated genes were mainly involved in the external encapsulating structure and cell wall.

### 2.5. Up-Regulation of Pollen Fertility-Related Genes in Huaduo1 Compared with Its Two Parents

**Figure 6:**
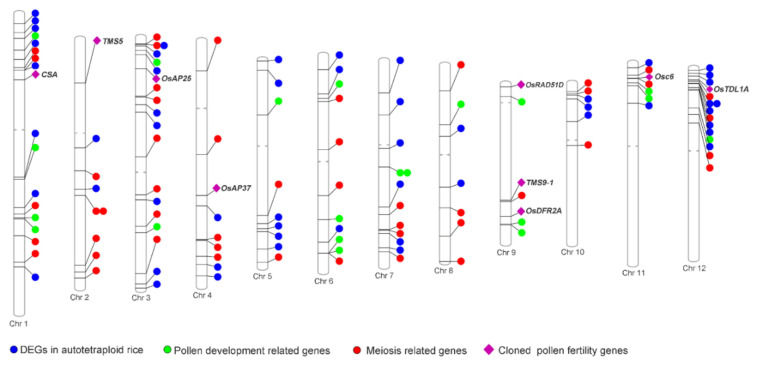
The distribution of up-regulated genes involved in meiosis and pollen development in H1 compared with its two parents. DEGs in autotetraploid rice indicated the DEGs detected in the pollen development process of autotetraploid rice; Pollen fertility genes indicated the DEGs involved in the pollen development process; Meiosis-related genes indicated the DEGs involved in meiosis; Cloned pollen fertility genes indicated the known pollen fertility DEGs.

To reveal the cause of higher pollen fertility in H1, we focused on the pollen fertility-related genes that were commonly differentially expressed and presented genetic variation in H1 through RNA-sequencing analysis. We compared our DEGs detected in H1 and its two parents with the large number of pollen fertility-related genes [10,25,26,27,28]. Of these DEGs, 193 genes were annotated as pollen fertility genes when combined with other analysis results. Notably, 129 of the pollen fertility genes were shown to be up-regulated in H1 compared with its two parents (Figure 6; Table S7). Predicted protein–protein interaction analysis was used to further evaluate the relationship of these pollen fertility genes. From this study, 37 of the up-regulated pollen fertility genes were predicted to be involved in a protein–protein interaction network in H1 (Figure S2). Among the 129 up-regulated genes, nine pollen fertility-related genes were demonstrated to cause low pollen fertility when down-regulated in previous research. For example, *LOC_Os01g16810*, *LOC_Os02g12290*, *LOC_Os03g08790*, *LOC_Os04g37570*, *LOC_Os09g01680*, *LOC_Os09g27620*, *LOC_Os09g32025*, *LOC_Os11g37280*, and *LOC_Os12g28750*, were detected as up-regulated in H1.

Among these genes, *OsRAD51D* (*LOC_Os09g01680)* is a DNA repair protein RAD51 homolog 4, and loss of function this gene results in pollen sterility [29]. *MIL2* (*LOC_Os12g28750*) regulates pollen fertility and cell differentiation in early pollen development [30,31]. *OsAP25* (*LOC_Os03g08790*) and *OsAP37* (*LOC_Os04g37570)* are aspartic proteinases and are directly regulated by *EAT1*, which plays an important role in the programmed cell death of tapetal cells in rice anthers [32]. *OsDFR2A* (*LOC_Os09g32025)* is an NAD-dependent epimerase/dehydratase family protein, which results in male sterility in rice [33]. *OsC6* (*LOC_Os11g37280*) encodes a lipid transfer protein and is essential to anther development [34]. *TMS9-1* (*LOC_Os09g27620*), also known as *PTC1* and *OsMS1*, is a transcript factor containing a plant homeodomain (PHD) finger domain that controls pollen sterility under high temperature [35,36,37].

Here, we also detected that two other genes, named *CSA* (*LOC_Os01g16810*) and *TMS5* (*LOC_Os02g12290*), were differentially expressed in H1 compared with its low-fertility-parent T44-4x. Both *CSA* and *TMS5* are pollen fertility-related genes that are also responsive to the environment. *CSA* (*LOC_Os01g16810*) is an *MYB* family transcription factor encoding an MYB protein domain that plays an important role in the pollen development process under short-day conditions [38]. *TMS5* (*LOC_Os02g12290*) is a nuclear ribonuclease Z that processes the mRNAs of three *ubiquitin fusion ribosomal protein L40* (*UbL40*) genes into multiple fragments, which could result in pollen sterility under high temperature [39].

To verify the expression profiles of pollen fertility genes in H1 and its two parents, nine representative pollen fertility-related genes were selected and validated by quantitative real-time reverse transcription PCR (qRT-PCR) analysis. All nine genes—*LOC_Os01g16810*, *LOC_Os02g12290*, *LOC_Os03g08790*, *LOC_Os04g37570*, LOC_Os09g01680, *LOC_Os09g27620*, *LOC_Os09g32025*, *LOC_Os11g37280*, and *LOC_Os12g28750*—were consistent with the transcriptome analysis. These results indicated that the expression levels of the nine genes were consistent with the transcriptome analysis, indicating the reliability and accuracy of RNA-sequencing results (Figure S3).

### 2.6. Knock-Out of Candidate Genes Causes Pollen Abortion in Huaduo1

To verify the function of upregulated pollen fertility genes in new tetraploid rice, CRISPR/Cas9 technology was used to conduct gene knockout in H1. In this study, we selected *TMS9-1* and *TMS5* as the representative pollen fertility genes, as these two genes were not only up-regulated in new tetraploid rice, but also down-regulated in autotetraploid rice compared with the original diploid rice in a previous study [10,15]. In the present work, we obtained at least 20 independently regenerated transgenic lines of *TMS5* and *TMS9-1* after the transformation (Figures S4 and S5). The mutant lines were grown in the field, and the T_2_ mutants were sequenced (Figures S4 and S5). We further selected the homozygous mutants of *TMS5* and *TMS9-1*, named *nt-tms5-1* and *nt-tms9-1*, to observe the pollen fertility and pollen development process.

**Figure 7:**
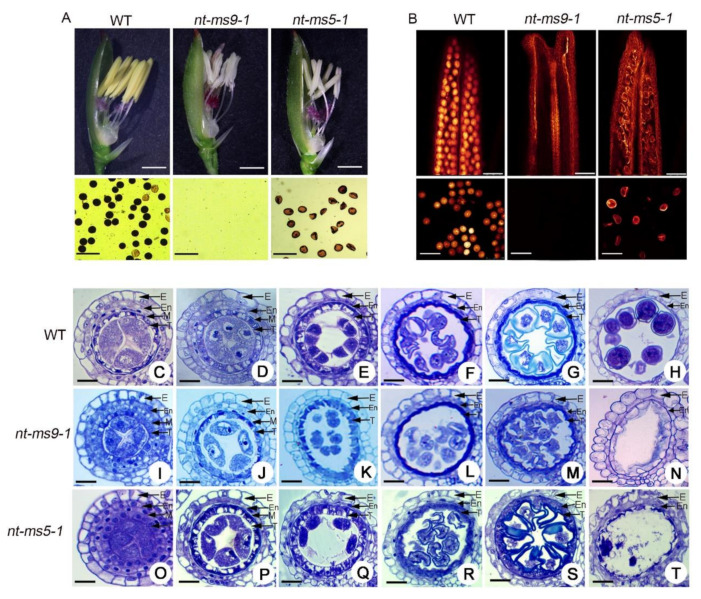
Phenotypic comparison and developing rice anthers between wild type (WT) and its two knock out lines (*nt-tms9-1* and *nt-tms5-1*) in H1. (**A**) Floral organs and pollen fertility between the wild type and its knock out lines after removal of the lemma. Bars = 1 mm. (**B**) Anthers and mature pollen grains between the wild type and its knock out lines using WE-CLSM analysis. Bars = 100 μm. C to H Semi-thin sections of wild type anthers. (**C**) pre-meiotic interphase; (**D**) meiosis stage I; (**E**) meiosis stage II; (**F**) single microspore stage; (**G**) bicellular stage; (**H**) mature pollen stage. I to N Semi-thin sections of *nt-tms9-1* anthers. (**I**) pre-meiotic interphase; (**J**) meiosis stage I; (**K**) meiosis stage II; (**L**) single microspore stage; (**M**) late bicellular stage; (**N**) mature pollen stage. O to T Semi-thin sections of *nt-tms5-1* anthers. (**O**) pre-meiotic interphase; (**P**) meiosis stage I; (**Q**) meiosis stage II; (**R**) single microspore stage; (**S**) late bicellular stage; (**T**) mature pollen stage. E, En, M and T indicate the epidermis, endothecium, middle layer, and tapetum, respectively. Bars = 50 μm.

In this study, both *nt-tms5-1* and *nt-tms9-1* showed marked differences in the anther and pollen compared with the wild type (WT) (Figure 7A). Pollen fertility in *TMS5* and *TMS9-1* knock-out lines showed higher pollen sterility, and the values were notably reduced compared with that of the wild type (Figure 7A). The statistical analysis results demonstrated that the pollen fertility values of *nt-tms5-1* and *nt-tms9-1* were much lower than that of the wild type. Moreover, whole-mount eosin B-staining confocal laser scanning microscopy (WE-CLSM) analysis of the anthers in *nt-tms5-1* and *nt-tms9-1* further verified that the number of normal pollen was clearly decreased compared with that of the wild type (Figure 7B).

Anther development was further investigated between the *nt-tms5-1*, *nt-tms9-1*, and the wild type. In the wild type, a four-layer anther wall (from the outside to the inside: epidermis, endothecium layer, middle layer, and tapetum) was generated at the pollen mother cell formation stage. No obvious defects were found between the WT and its knock out lines in the formation of PMCs (Figure 7C,I,O). During pollen mother cell (PMC) meiosis, dyads and tetrads were normally formed in the WT (Figure 7D,E) and its knockout lines (Figure 7J,K,P,Q). Thereafter, the microspores of WT underwent vacuolation and mitosis to form mature pollen with spherical or elliptical shapes (Figure 7F–H). In contrast, the microspores of two knock out lines (*nt-tms5-1* and *nt-tms9-1*) degraded further after the late microspore stage and completely disappeared at the mature pollen stage, which resulted in an empty anther locule (Figure 7L–N,R,S). All of these results suggest that the lack of *TMS9-1* and *TMS5* also caused defects in the microspores as well as abnormal pollen in H1 during pollen development.

**Figure 8:**
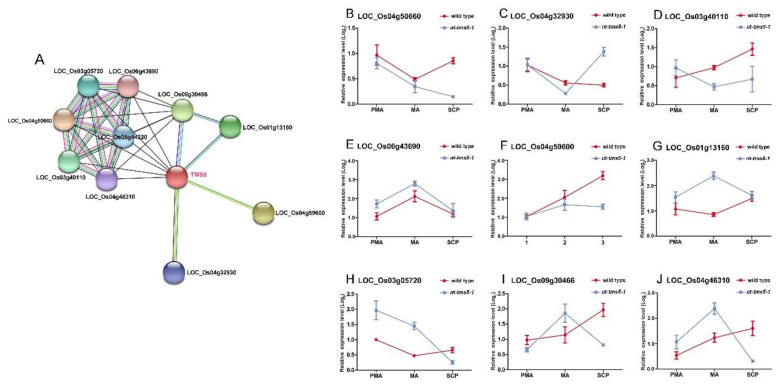
Verification of the predicted *TMS5* regulation network during the pollen development process in *nt-tms5-1* and its wild type. (**A**) Predicted protein–protein interaction network of the *TMS5* gene. Colored lines indicate the interaction proved by experiments, and grey lines indicate the predicted interaction. (**B**–**D**,**F**), indicate a lower gene expression level in *nt-tms5-1* compared with its wild type. (**E**,**G**–**J**), indicate a higher gene expression level in *nt-tms5-1* compared with its wild type. PMA, the pre-meiosis stage. MA, the meiosis stage. SCP, single microspore stage.

To further investigate if the knockout line regulated the pollen development process of H1, we used nt-*tms5-1* for further analysis (Figure 8A). From this study, nine genes were selected to analyze their gene expression levels in *nt-tms5-1* and its wild type (Figure 8B–J). We conducted qRT-PCR analysis to detect the nine transcripts of these genes in *nt-tms5-1* and the wild type (Figure 8). In this study, the expression levels of the nine genes were shown to be differentially expressed between *nt-tms5-1* and the wild type. For example, four genes, *LOC_Os04g50600*, *LOC_Os04g32930*, *LOC_Os03g40110*, and *LOC_Os04g59600*, were down-regulated in the meiosis stage in *nt-tms5-1* compared to their levels in the wild type. Five genes—*LOC_Os06g43690*, *LOC_Os03g13160*, *LOC_Os03g05720*, *LOC_Os09g30486*, and *LOC_Os04g46310*—were up-regulated in meiosis in *nt-tms5-1* compared to the wild type. These results verify that *TMS5* interacted with other genes and knockout of *TMS5* could also cause down-regulation of other genes in H1.

## 3. Discussion

### 3.1. Significant Phenotypic Variation Exists in Huaduo1 Compared with Its Two Parents

Autotetraploid rice is a new germplasm resource derived from diploid rice by chromosome doubling. Numerous advantages, such as a stronger stem, wider leaf, and larger grains, exist in autotetraploid rice compared to its diploid counterparts [9,10,40]. Agronomic traits in autotetraploid rice demonstrated significant potential to improve the rice biomass yield [9,38,41]. However, the lower fertility of autotetraploid rice is still an important issue for utilizing its potential vigor. It took us more than twenty years to generate the new tetraploid rice, and we found that it was one type of stable autotetraploid rice line derived from the progeny of autotetraploid rice [18,19]. In our previous analysis, we proposed that new tetraploid rice had higher fertility and hybrid vigor, which could overcome the low fertility of autotetraploid rice [18,19]. Therefore, it is of greater value to evaluate the phenotypic variation of the new tetraploid rice compared with its two parents.

In the present work, one of the newly developed new tetraploid rice lines, named H1, which has been registered for PVP in China, was used. We analyzed the phenotypic variation of H1 and detected that three of the seven primary agronomic traits, including the plant height, seed set ratio and 1000-grain weight, varied significantly compared to those of the two parents. Notably, the seed set ratio in H1 could reach >80%, which is much higher than that of the two parents. These obvious phenotypic variations are similar to the other type of new tetraploid rice [19] and show great potential for the utilization of H1. Additionally, we also evaluated the heterosis and gene interaction effect using Taichung 65-4x and its pollen sterility near-isogenic lines and found that the seed set ratio of the hybrids exceeded 80%. These results show the significant potential that H1 may have given the neutral genes for pollen fertility that could overcome the sterility of hybrids. The neutral genes could overcome the hybrid’s sterility caused by the multi-pollen sterility loci interactions in autotetraploid rice hybrids [14,42].

Pollen fertility was thought to be the important factor for determining production in autotetraploid rice. An abnormal pollen development process, meiotic chromosome behavior, microtubules or interactions of pollen sterility-related genes are the primary reasons leading to pollen abortion in autotetraploid rice [10,12,15]. Therefore, we observed the anther development and chromosome behavior of PMCs to detect the genetic variations between H1 and its parents. The results indicate that the percentage of abnormalities in PMC cells was higher in T44-4x and T45-4x compared with that in the H1. For example, the percentages of abnormal cells in T44-4x were 52.14%, 15.05%, 3.96%, 23.10%, 52.14%, and 12.12% in Metaphase I, Anaphase I, Telophase I, Metaphase II, Anaphase II, and Telophase II, respectively. In contrast, the percentages of abnormal cells in H1 were much lower than those of its two parents. Additionally, one interesting phenomenon was found in the configuration of H1 at the diakinesis stage. The configuration in H1 was primarily bivalent and quadrivalent and showed significant differences from its two parents. All of these results demonstrate that H1 showed obvious variation and a higher percentage of fertility than its two parents.

### 3.2. Upregulation of Pollen Fertility Genes May a Play Critical Role in the High Pollen Fertility in Huaduo1

New tetraploid rice exhibits higher fertility and greater heterosis compared with autotetraploid rice lines [18,19,22]; however, there are still limitations to understanding the high fertility mechanism of new tetraploid rice. Here, we used RNA-sequencing analysis to analyze the transcriptional variations between H1 and its two parents. Using the transcriptome analysis profile, we identified 440 common DEGs that showed at least a twofold change in H1 compared with its two parents. Gene ontology (GO) analysis was conducted to annotate the up- and down-regulated genes that were differentially expressed in H1 and its parents in this study. The GO enrichment classification indicated that DEGs in the cellular component, molecular function, and biological process categories showed significant variations. We found that up-regulated differentially expressed genes were mainly involved in the transport, transcription regulator activity-related, and reproduction processes.

Pollen development is an important reproductive process in autotetraploid rice, where errors can result in low fertility. Meiosis-related and pollen fertility genes were frequently detected to be downregulated, and this was thought to be the primary cause of the lower pollen fertility in autotetraploid rice [10,14,15,16]. Compared with autotetraploid rice, the pollen fertility and seed set of new tetraploid rice are much higher and exhibit normality [18,19]. In the present work, we combined our results with the pollen fertility genes detected in previous analyses [13,25,26,27,28,43]. A total of 240 pollen fertility genes were detected and found to be upregulated in H1 compared with its two parents. Notably, 79 of these pollen fertility genes were previously detected to be down-regulated in autotetraploid rice when compared with the corresponding diploid rice [10,13].

Therefore, we speculated that some important pollen fertility-related genes in the new tetraploid rice may be expressed normally again, resulting in high fertility in H1. In this study, nine cloned pollen fertility genes were up-regulated and found to be involved in meiosis, tapetum regulation, and environmentally sensitive genic male sterility [29,30,31,32,33,34,35,36,37]. For example, *OsRAD51D* (*LOC_Os09g01680)* regulates the DNA repair protein RAD51 homolog 4, and the loss of function of this gene results in pollen sterility [29]. The up-regulation of Os*RAD51D* observed in our study may promote normal chromosome pairing, resulting in a lower percentage of micronuclei in new tetraploid rice. Three tapetum related genes, named *MIL2* (*LOC_Os12g28750*), *OsAP25* (*LOC_Os03g08790*), and *OsAP37* (*LOC_Os04g37570*), were also up-regulated in new tetraploid rice. The loss of function of these genes resulted in pollen sterility and the abnormal programmed cell death process of tapetal cells. In the case of autotetraploid rice, abnormalities in tapetal cells were frequently observed and were a primary cause of the low pollen fertility [15].

The up-regulation of tapetum-related genes in our study may promote abnormalities in tapetal cells, resulting in high pollen fertility in new tetraploid rice. Additionally, three genes, named *CSA* (*LOC_Os01g16810*), TMS5 (*LOC_Os02g12290*), and *TMS9-1* (*LOC_Os09g27620*), are pollen fertility-related genes that are also responsive to the environment. *CSA* (*LOC_Os01g16810*) is an *MYB* family transcription factor that encodes an *MYB* protein domain, which plays an important role in the pollen development process. *TMS5* (*LOC_Os02g12290*) is a nuclear ribonuclease Z that processes the mRNAs of three ubiquitin fusion ribosomal protein L40 (UbL40) genes into multiple fragments, which could result in pollen sterility under low temperatures. *TMS9-1* (*LOC_Os09g27620*), also known as *PTC1* and *OsMS1*, is a male sterility gene and could result in pollen sterility [35,36,37]. All of these results were not only verified by our transcriptome analysis data but also indicated that pollen fertility genes may play an important role in H1.

The down-regulation of pollen fertility-related genes or meiosis-related genes was thought to be the primary cause of low pollen fertility in autotetraploid rice [10,15]. Several studies indicated that these genes were down-regulated in autotetraploid rice compared with the original diploid rice [10,11,15]. Compared with autotetraploid rice, high pollen fertility is a primary characteristic of H1. We speculated that some important pollen fertility-related genes may be expressed normally again and may have a relationship with the pollen development process. Therefore, we focused on up-regulated DEGs in the new tetraploid rice compared with autotetraploid rice.

With the advantage of effective tools such as CRISPR/Cas9 technology, we had the opportunity to knock out important fertility-related genes. In this study, we selected two up-regulated genes, *TMS5* and *TMS9-1*, to evaluate the role of pollen fertility genes in H1. Notably, two genes were also detected as down-regulated in autotetraploid rice compared with the original diploid rice [10,15]. Our knockout results indicated that *TMS5* and *TMS9-1* play an important role in pollen fertility and resulted in pollen sterility in H1. Compared with the wild type, *nt-tms5* and *nt-tms9-1* exhibited marked differences in anther, panicle, and pollen fertility values. All of these results verify our speculation and indicate that the up-regulation of pollen fertility genes may play critical roles in the high pollen fertility of H1. In addition, as *TMS9-1* and *TMS5* are important pollen sterility genes, this method also provides a possibility for boosting the development of excellent pollen sterility lines and further revealing the mechanism of high pollen fertility in H1. In the future, better yield and quality can also be developed by editing important pollen fertility genes for fertility and disease at the same time in H1 with the appropriate genetic background.

## 4. Materials and Methods

### 4.1. Plant Material

Three materials, Huaduo1 (H1) and its two parents (Jackson-4x and 96025-4x), were used in this study. Jackson-4x (T45-4x) is autotetraploid rice, produced by artificial polyploidization with colchicine. 96025(T44-4x) is an autotetraploid rice line. H1 is the high-pollen-fertility material derived from the hybrid cross between T45-4x (maternal) and T44-4x (paternal) followed by self-crossing for more than 25 generations in our laboratory. All of these materials were planted under natural conditions at the experimental farm of South China Agricultural University (SCAU), and our standard practices followed the recommendations for the area.

### 4.2. Analysis of Agronomic Traits and Heterosis

To detect the genetic variation of H1 and its two parents, a total of eight agronomic traits were selected to detect the phenotypic variation, i.e., the plant height (PH, cm), panicle length (PL, cm), effective panicle number (EPN), panicle length (PL, cm)**,** total number of grains per plant (TGP), 1000-grain weight (GWT, g), and seed set ratio (SS = (number of filled grains/total number of grains) × 100). These traits were detected according to our previous study [9].

Heterosis analysis was conducted to evaluate the heterosis level of H1, with a total of 13 differential parents crossed with the H1 and F_1_ hybrid. The high-parent heterosis (HPH) and mid-parent heterosis (MPH) were estimated by the following formula: HPH = (F_1_ − HP)/HP × 100%, and MPH = (F_1_ − MP)/MP × 100%, where F_1_ is the performance of the first filial generation (hybrid), HP is the performance of the best parent, and MP is the average performance of two parents.

### 4.3. Pollen Fertility, Semi-Thin Section Analysis

The pollen fertility of H1 and its two parents was observed according to our previous study with minor modifications [44]. More than 1000 pollen grains were evaluated for pollen fertility under a microscope (Motic BA200, Lampertheim, Germany).

Semi-thin section analysis was performed according to Li et al. (2018) [15]. The samples were fixed in formalin–acetic acid–alcohol (FAA) solution for 48 h. After being washed in 50% ethanol several times, the samples were dehydrated in a series of ethanol solutions and then embedded by a Leica 7022 historesin embedding kit (Leica, Nussloch, Germany) according to the manufacturer’s instructions. The embedded samples were further sectioned using a Leica RM2235 manual rotary microtome, stained with 1% toluidine blue O, and sealed with neutral balsam. The detailed procedures were described previously [15].

### 4.4. Chromosome Behavior Observation

The meiosis chromosome behavior experiment was performed according to Wu et al. (2014) [10]. To observe the chromosome behavior in the meiosis process, samples were collected from the shoots of rice plants with −2 to 2 cm between their flag leaf cushion and the second-to-last leaf cushion. Then, the samples were fixed in Carnoy’s solution (ethanol/acetic acid, 3:1 *v*/*v*) for at least 24 h and were washed using 95% and 80% ethanol for ~30 min each. Finally, they were washed and kept in 70% ethanol at 4 °C until observation.

### 4.5. WE-CLSM Analysis

The WE-CLSM analysis was performed according to Zeng et al. (2007) [45]. Anthers and mature pollens were stained using a small drop of 10 mg/L eosin B (C20H6N2O9Br2Na2, FW 624.1, a tissue stain for cell granules and nucleoli) solution (dissolved in 4% sucrose) on a glass slide. After 10 min, the glass slide was covered with a slide cover and scanned under a Leica SP2 laser scanning confocal microscope (Leica Microsystems, Heidelberg, Germany). The detailed procedures were described previously [43].

### 4.6. Sample Preparation, RNA Extraction, RNA-Sequencing, and qRT-PCR Verification

Anthers (H1, T44-4x and T45-4x) from the pre-meiotic stage to Prophase I were collected from three biological replicates and stored at −80 °C until RNA extraction. The total RNA of each sample was extracted using Trizol reagent (Invitrogen, CA, USA) following the manufacturer’s procedure. The quantity and purity of the total RNA were analyzed using a Bioanalyzer 2100 and RNA 6000 Nano Lab Chip Kit (Agilent, CA, USA) with RIN number > 7.0. The RNA-sequencing library preparation was carried out according to the manufacturer’s protocol and was performed on an Illumina HiSeqTM2500 by Biomarker Technologies (Beijing, China). Genes with FC ≥ 2 (fold change) and false discovery rate (FDR) ≤ 0.01 were chosen for the t-test, and genes with *p* values < 0.05 were chosen for further analysis. After selecting the differentially expressed genes, cluster analysis and GO enrichment analysis were conducted using Cluster 3.0 software (Tokyo, Japan ) and agriGO 2.0 (Beijing, China). Venny 2.1 software (BioinfoGP, Madrid, Spanish) was used to identify the overlapped differentially expressed genes in different samples (http://bioinfogp.cnb.csic.es/tools/venny/). Predicted protein–protein interactions were analyzed using STRING website (http://www.string-db.org/).

Real-time qRT-PCR analysis was conducted to examine the expression patterns of H1 and its two parents. A total of nine candidate genes were selected and used to validate the transcriptome data using the same RNA samples of RNA-sequencing (Table S8a). A reverse transcription reaction was performed using the Roche Transcriptor First Strand cDNA Synthesis kit. The qRT-PCR experiment was performed on a Lightcycler480 system (Roche, Basel, Switzerland) using the Advanced SYBR Green Supermix Kit (Bio-RAD, Hercules, CA, USA). The qRT-PCR cycles had the following reaction conditions: 95 °C for 30 s, 40 cycles of 95 °C denaturation for 5 s, and 58 °C annealing and extension for 20 s. All qRT-PCR reactions were performed in triplicate, and the results were calculated using the 2^−ΔΔCt^ method [46]. The rice ubiquitin gene was selected and used as an internal control to normalize the expression levels [10]. Each PCR was repeated three times.

### 4.7. Generation and Mutation Sequencing of Mutant Plants in Huaduo1

We used the CRISPR-Cas9 binary vector pC1300-Cas9 to knock out *TMS9-1* and *TMS5* to obtain the *nt-*t*ms9-1 and nt-tms5-1* mutants. Genome sequences of TMS9-1 and TMS5 in H1 were used to design the target sequences using the target design tool of CRISPR-GE (http://skl.scau.edu.cn/targetdesign/) [47]. The target-site primers were located at the start of the exons to cause frameshift mutations. The CRISPR/Cas9 vectors were constructed as described previously [48]. These vectors were introduced to EHA105 and transformed into H1 to generate the nt-tms9-1 and nt-tms5-1 mutant lines. We extracted the genomic DNA from transformants, and the genomic DNA was sequenced for mutant identification. The PCR products (500–800 bp) were sequenced and identified using the de-generate sequence decoding method [47]. Mutations were identified by comparing the amplicon sequences derived from putative transgenic and pC1300-cas9 templates.

## 5. Conclusions

In the present study, we found that Huaduo1 (H1) displayed higher pollen fertility, a lower percentage of pollen mother cell abnormalities, and more stable chromosome configurations during the pollen development process. Our results provide evidence that up-regulation of the pollen fertility genes plays an important role in the high fertility of new tetraploid rice. We detected 129 up-regulated pollen fertility genes that can be used as candidate genes to reveal the mechanism of high pollen fertility in new tetraploid rice in the future.

## Figures and Tables

**Table 1:** Genetic variation in agronomic traits of neo-tetraploid rice and its parents.

Seasons	Material Name	PH (cm)	EPN	PL (cm)	FG	TGP	SS (%)	GWT (g)
Late season	T45-4x	94.40 ± 1.19 **	3.40 ± 0.27 **	25.97 ± 0.60	63.27 ± 4.78	120.90 ± 9.17 *	53.27 ± 2.34 **	30.74 ± 1.39 *
	T44-4x	78.10 ± 1.58 **	5.65 ± 0.44	23.53 ± 0.33	15.58 ± 1.41 **	55.89 ± 1.94 *	26.45 ± 1.88 **	31.39 ± 0.46
	H1	104.40 ± 0.93	6.60 ± 0.40	25.46 ± 0.52	66.34 ± 4.63	87.02 ± 6.34	76.46 ± 1.43	35.44 ± 0.34
Early season	T45-4x	117.70 ± 0.64 **	4.45 ± 0.28	31.74 ± 0.46 **	40.26 ± 1.33 **	132.38 ± 4.05	30.80 ± 0.97 **	30.82 ± 1.04 **
	T44-4x	96.05 ± 0.46 **	5.85 ± 0.27 *	28.81 ± 0.34	30.17 ± 0.65 **	77.90 ± 0.55 **	39.16 ± 7.16 **	33.74 ± 0.49
	H1	124.45 ± 0.84	4.95 ± 0.22	28.09 ± 0.24	92.75 ± 3.57	120.41 ± 4.23	76.55 ± 0.84	33.86 ± 0.29

Note: *, ** Significantly different from zero at *p* < 0.05 and *p* < 0.01, respectively. PH = plant height, EPN = effective panicle number, PL = panicle length, FG = filled grains, TGP = total number of grains per plant, GWT = 1000-grain weight and SS = seed set ratio.

**Table 2:** Pollen fertility of neo-tetraploid rice and its parents.

Material Name	Pollen Fertility (%±SE)	Typical Aborted Pollen (%±SE)	Stained Aborted Pollens (%±SE)	Small Pollen (%±SE)
T45-4x	54.10 ± 3.81	8.18 ± 0.72	37.26 ± 3.92	0.46 ± 0.12
T44-4x	57.66 ± 2.78	15.08 ± 1.56	23.07 ± 1.98	4.19 ± 0.51 **
H1	95.62 ± 0.34 **	3.16 ± 0.34 **	0.60 ± 0.13 **	0.62 ± 0.12

Note: ** indicates a significant difference in pollen fertility between neo-tetraploid rice and its parents at *p* < 0.01.

## Supplementary Materials

The following are available online at https://www.mdpi.com/1422-0067/21/19/7046/s1, Figure S1: GO analysis of common differentially expressed genes in H1 comparative its two parents. Figure S2: Predicted protein-protein interaction network of pollen fertility genes in H1 compared with its parents. Figure S3: Comparison of the log2 (FC) of nine selected genes using qRT-PCR analysis. Figure S4: Mutations of *TMS9-1* target sites and its expression level in H1 and *nt-tms9-1*. Figure S5: Mutations of *TMS5* target sites and its expression level in H1 and *nt-tms5*. Table S1: Heterosis analysis of hybrids generated by the crossing of H1 and autotetraploid rice lines. Table S2: Seed set ratio of hybrids with genetic interactions at Sa, Sb and Sc loci. Table S3: Frequency of abnormal chromosome behaviors in H1 and its parents during the Meiosis I. Table S4: Frequency of abnormal chromosome behaviors in H1 and its parents during the Meiosis II. Table S5: Meiotic chromosome configurations in H1 and its parents. Table S6a: Differentially expressed genes in H1 derived from T44-4x. Table S6b: Differentially expressed genes in H1 derived from T45-4x. Table S6c: Common differentially expressed genes in H1 compared with its parents. Table S7: Functional pollen fertility related genes in H1 compared with its two parents. Table S8a: List of primers used for qRT-PCR analysis. Table S8b: List of primers used for verify the gene expression in nt-tms5 and wild type.

## Abbreviations

PMC	Pollen mother cell
DEGs	Differentially expressed genes
PVP	Protection for new varieties of plants
GO	Gene ontology
TIGR	The Institute for Genomic Research Rice Genome Annotation
GEO	Gene Expression Omnibus
WE-CLSM	Whole-mount eosin B-staining confocal laser scanning microscopy
UbL40	Ubiquitin fusion ribosomal protein L40
PH	Plant height
PL	Panicle length
EPN	Effective panicle number
TGP	Total number of grains per plant
SS	Seed set ratio
HPH	High-parent heterosis
MPH	Mid-parent heterosis
FAA	Formalin–acetic acid–alcohol
PCR	Polymerase chain reaction
