# Supplementary material for: Comparative Cytological and Transcriptome Analysis Revealed the Normal Pollen Development Process and Up-Regulation of Fertility-Related Genes in Newly Developed Tetraploid Rice
Source: Int J Mol Sci. 2020 Sep 24;21(19):7046. doi: 10.3390/ijms21197046 (PMC7582553; doi:10.3390/ijms21197046)
Supplement: Supplementary file 1 [file ijms-21-07046-s001.zip › Supple mental file1 figures.pdf]

A. Biological process

GO Description	Up-regulation	Down-regulation
cellular process	2.33E-05	
transport	4.20E-02	
transcription	3.21E-03	
reproductive process	4.70E-05	
reproduction	3.99E-02	
pollination	3.58E-02	
response to stimulus		1.65E-03
response to stress		3.22E-03
response to endogenous stimulus		4.40E-03

B. Cell component

GO Description	Up-regulation	Down-regulation
nucleus	1.46E-02	
ntegral to membrane	4.55E-02	
intrinsic to membrane	4.57E-02	
extracellular region		2.27E-02
cell wall		1.89E-02
external encapsulating structure		4.43E-02
thylakoid		1.22E-02

C. Molecular function

GO Description	Up-regulation	Down-regulation
transcription regulator activity	9.75E-03	
DNA binding	1.09E-02	
transporter activity	2.70E-02	
cation binding	4.55E-02	
catalytic activity		7.32E-03
ion binding		1.27E-02
oxidoreductase activity		1.83E-02
nucleotide binding		2.58E-02

Figure S1. GO analysis of common differentially expressed genes in H1 comparative its two parents.

Note: Genes were divided into three categories: biological process, cell component, and molecular function. **A** Significant GO terms of biological process category between up-regulation and down regulation genes. **B** Significant GO categories of cell component category between up-regulation and down regulation genes. **C** Significant GO categories of molecular function category between up-regulation and down regulation genes. P value was listed in the significant GO categories.

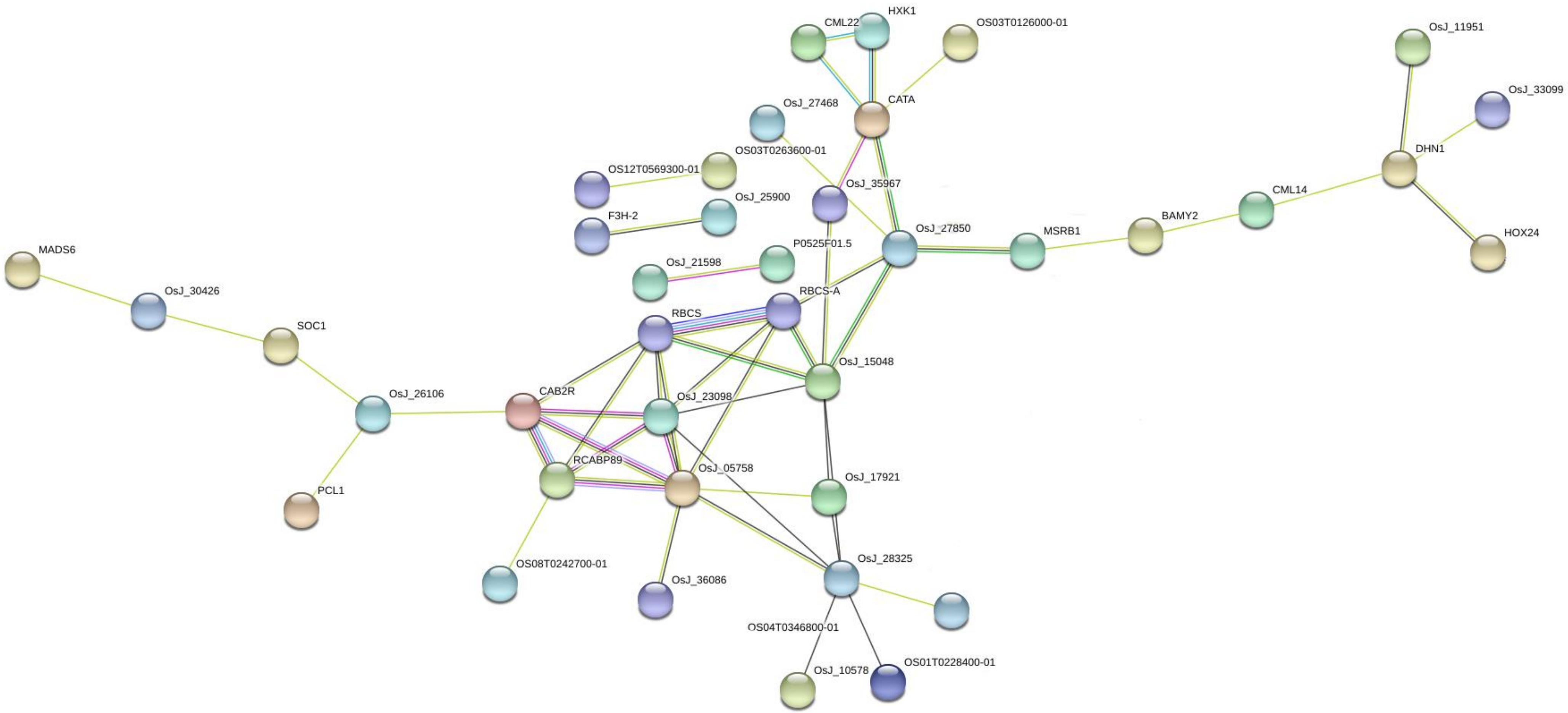

Figure S2. Predicted protein-protein interaction network of pollen fertility genes in H1 compared with its parents

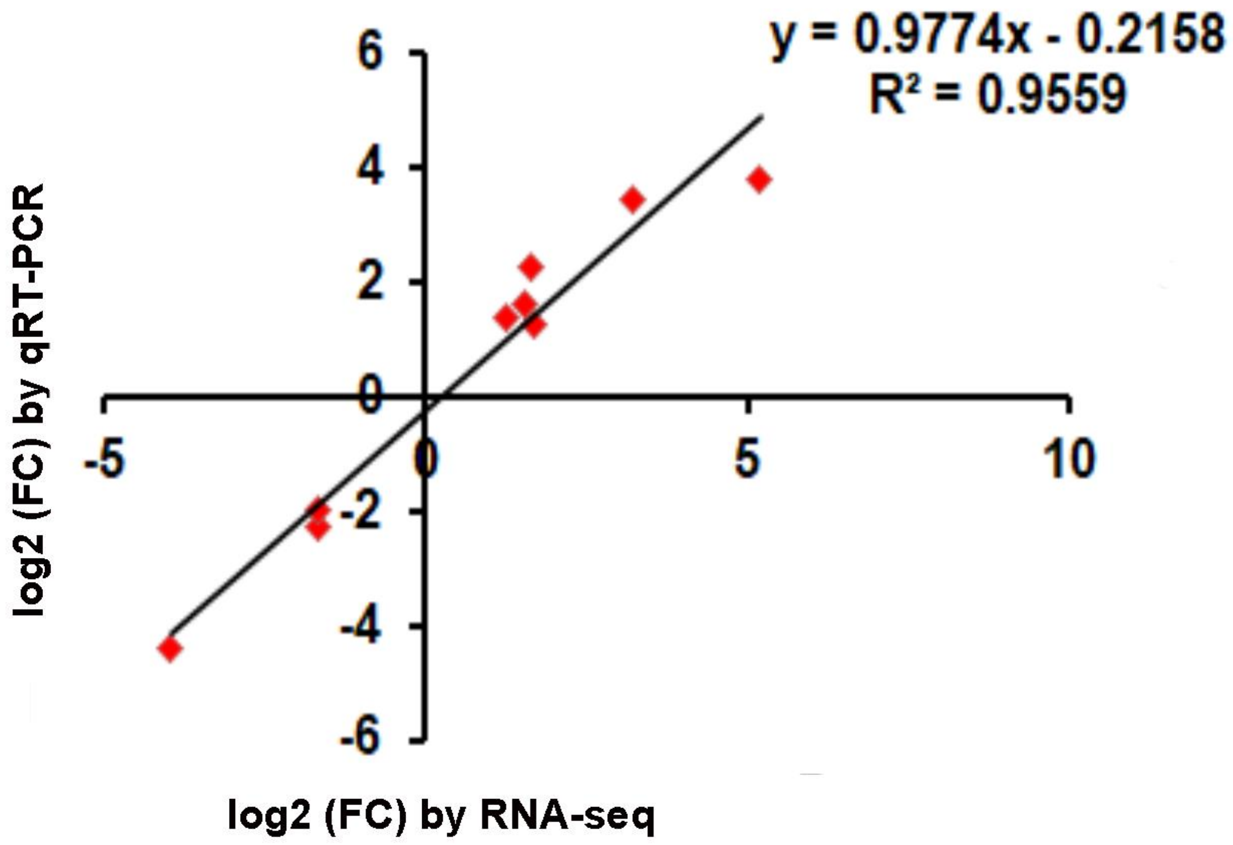

Figure S3. Comparison of the $\log_2$ (FC) of nine selected genes using qRT-PCR analysis.

Note: Both the $\log_2$ transformed values of relative expression of qRT-PCR and fold changes of RNA-seq data were used for linear regression. The Pearson's correlation coefficient was used to measure the linear correlation of qRT-PCR and RNA-seq analysis.

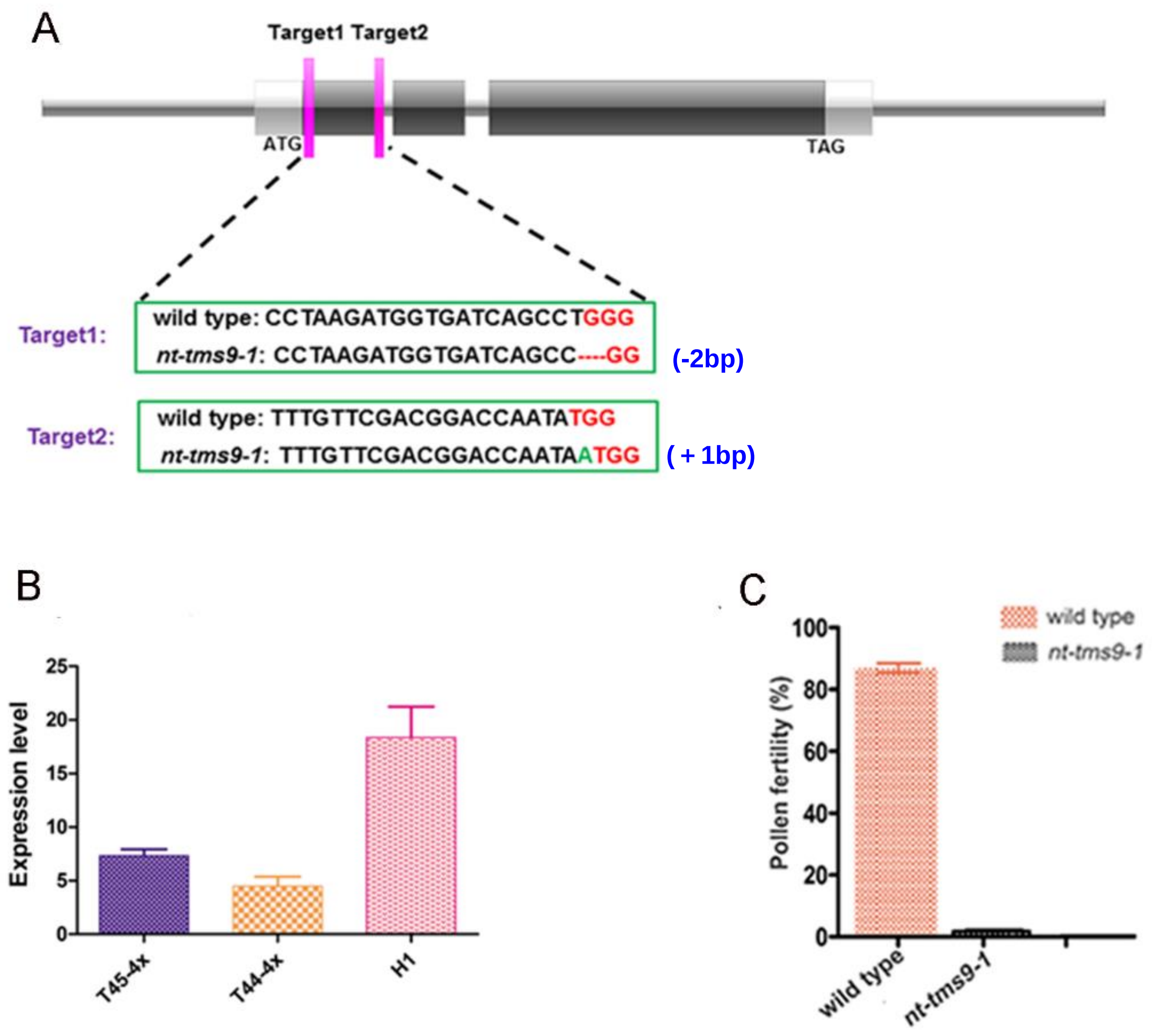

Figure S4 Mutations of *TMS9-1* target sites and its expression level in H1 and *nt-tms9-1*

Note: **A** PAM sequence and two target sites of *TMS9-1* in neo-tetraploid rice. **B** Expression level of *TMS9-1* in neo-tetraploid rice and its two parents. **C** Pollen fertility of *TMS9-1* knockout lines (*nt-tms9-1*) and its wild type.

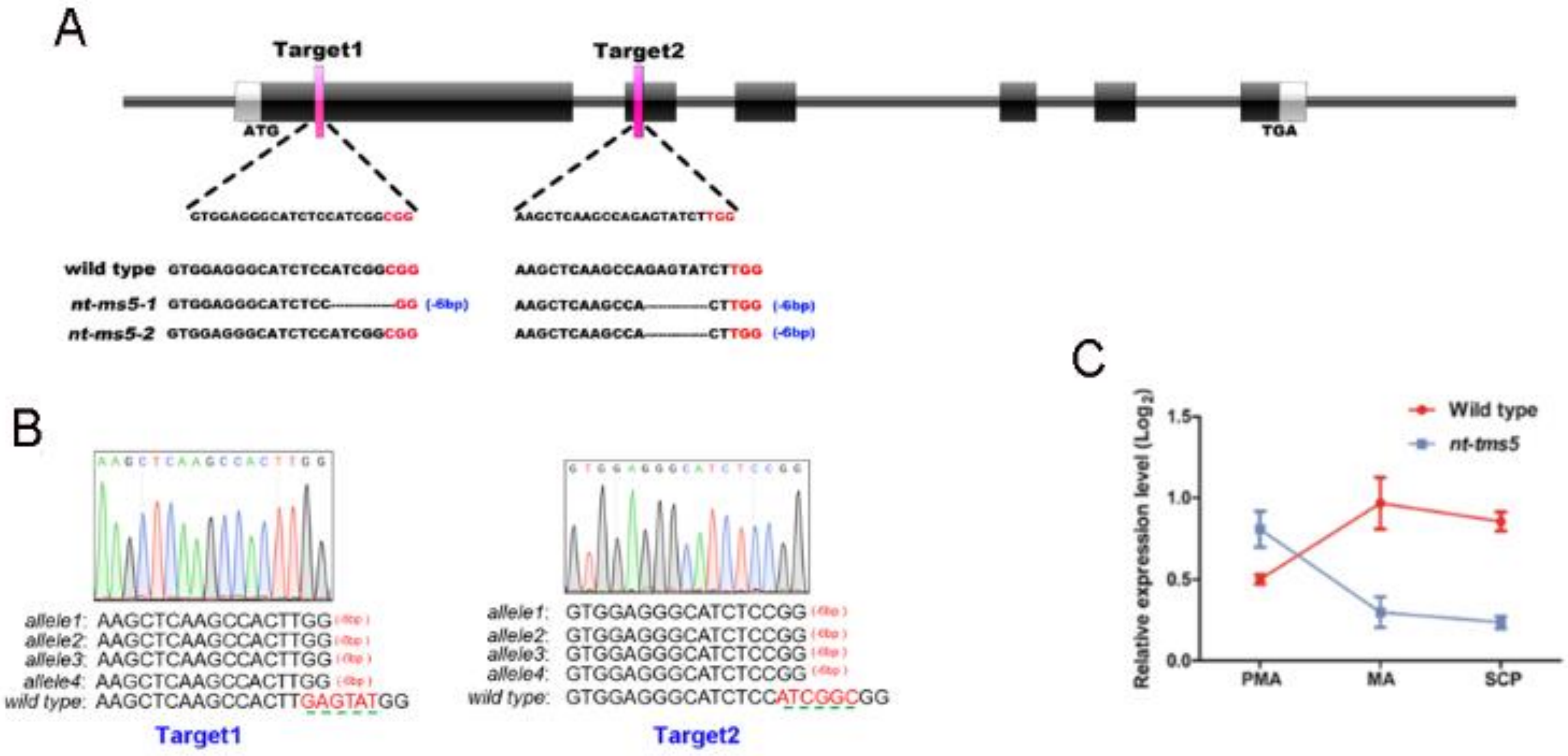

Figure S5 Mutations of *TMS5* target sites and its expression level in H1 and *nt-tms5*

Note: **A** PAM sequence and two target sites of *TMS5* in neo-tetraploid rice. **B** Sequencing results of two targets in *TMS5* knockout lines. **C** Expression analysis of TMS5 in its wild type and *nt-tms5* mutant.
